# Listeriosis Caused by Persistence of *Listeria monocytogenes* Serotype 4b Sequence Type 6 in Cheese Production Environment

**DOI:** 10.3201/eid2701.203266

**Published:** 2021-01

**Authors:** Magdalena Nüesch-Inderbinen, Guido V. Bloemberg, Andrea Müller, Marc J.A. Stevens, Nicole Cernela, Beat Kollöffel, Roger Stephan

**Affiliations:** Institute for Food Safety and Hygiene, Zurich, Switzerland (M. Nüesch-Inderbinen, M.J.A. Stevens, N. Cernela, R. Stephan);; National Reference Centre for Enteropathogenic Bacteria and *Listeria*, Zurich (G.V. Bloemberg, A. Müller);; Kantonales Laboratorium der Urkantone, Brunnen, Switzerland (B. Kollöffel)

**Keywords:** outbreak, listeriosis, Listeria monocytogenes, bacteria, serotype 4b, sequence type 6, ST6, hypervirulent emerging clone, cheese, cheese production environment, contamination, persistence, food safety

## Abstract

A nationwide outbreak of human listeriosis in Switzerland was traced to persisting environmental contamination of a cheese dairy with *Listeria monocytogenes* serotype 4b, sequence type 6, cluster type 7488. Whole-genome sequencing was used to match clinical isolates to a cheese sample and to samples from numerous sites within the production environment.

Listeriosis is a potentially lethal infection, and the elderly population, pregnant women, and immunocompromised persons at particular risk (*1*). Foods, in particular ready-to-eat foodstuffs, including meat, fish, dairy products, fruits, and vegetables, represent the major vehicle for sporadic cases and outbreaks of listeriosis (*2*). *Listeria monocytogenes* serotype 4b sequence type 6 (ST6) has emerged since 1990 as a hypervirulent clone that is associated with particularly worse outcome for case-patients who have *Listeria* meningitis and therefore poses a particular threat to consumer health (*3**,**4*).

*L. monocytogenes* ST6 is increasingly associated with outbreaks, including an outbreak linked to frozen vegetables in 5 countries in Europe during 2015–2018 (*5*), an outbreak associated with contaminated meat pâté in Switzerland during 2016 (*6*), and the largest listeriosis outbreak globally, which occurred in South Africa during 2017–2018 (*7**,**8*). More recently, the largest outbreak of listeriosis in Europe in the past 25 years was reported in Germany and was traced back to blood sausages contaminated with *L. monocytogenes* ST6 belonging to a particular clone referred to as Epsilon1a (*9*).

Human listeriosis is a reportable disease in Switzerland. All cases of culture- or PCR-confirmed human listeriosis are reported to the Swiss Federal Office of Public Health (SFOPH). Diagnostic laboratories and regional (cantonal) laboratories forward isolates to the Swiss National Reference Centre for Enteropathogenic Bacteria and *Listeria* for strain characterization, ensuring early recognition of *Listeria* clusters among food isolates or human cases. We report an outbreak of listeriosis associated with cheese contaminated with *L. monocytogenes* 4b ST6 in Switzerland.

## The Study

In 2018, the SFOPH recorded 52 human cases of listeriosis, corresponding to a normal annual incidence rate of 0.6 cases/100,000 inhabitants (*10*). However, during March 6, 2018–July 31, 2018, an increase of *L. monocytogenes* serotype 4b from 13 human cases was recorded. Whole-genome sequencing (WGS) was performed on these strains by using MiSeq next generation sequencing technology (Illumina, https://www.illumina.com). Sequencing reads were mapped against an mutlilocus sequencing typing (MLST) scheme based on 7 housekeeping genes and a 1,701-locus core genome MLST (cgMLST) scheme by using Ridom SeqSphere+ software version 5.1.0 (*11*). STs and cluster types (CTs) were determined upon submission to the *L. monocytogenes* cgMLST Ridom SeqSphere+ server (https://www.cgmlst.org/ncs/schema/690488/).

A cluster was defined as a group of isolates with <10 different alleles between neighboring isolates (*9**,**11*). Twelve of 13 isolates were assigned to ST6 CT7448, a unique profile in the database, showed by cluster detection to be closely related. Accordingly, we defined an outbreak case-patient as a patient who had listeriosis and *L. monocytogenes* ST6 CT7448. An outbreak investigation was initiated by the SFOPH, and patients were contacted to assess food exposures by using a standardized questionnaire. Diagnostic and cantonal laboratories were notified nationwide to ensure rapid submission of *L. monocytogenes* isolates to the National Reference Centre for Enteropathogenic Bacteria and *Listeria* for laboratory typing, including WGS. However, the questionnaire-based outbreak investigation did not lead to a suspect food, and the vehicle of infection remained unknown.

**Figure 1:**
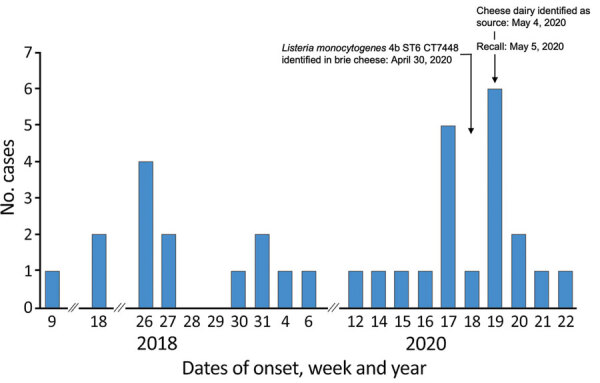
Cases of human listeriosis caused by *Listeria monocytogenes* ST6 CT7488, by week and year, Switzerland, 2018 and 2020. CT, cluster type; ST, sequence type.

In a second wave, onset dates ranged from January 22 to May 26, 2020 (Figure 1). Another 27 cases of infection with *L. monocytogenes* serotype 4b were recorded; 4 cases were in hospital patients who had underlying conditions. During this period, questionnaire-based data were not available to support a food hypothesis.

**Figure 2:**
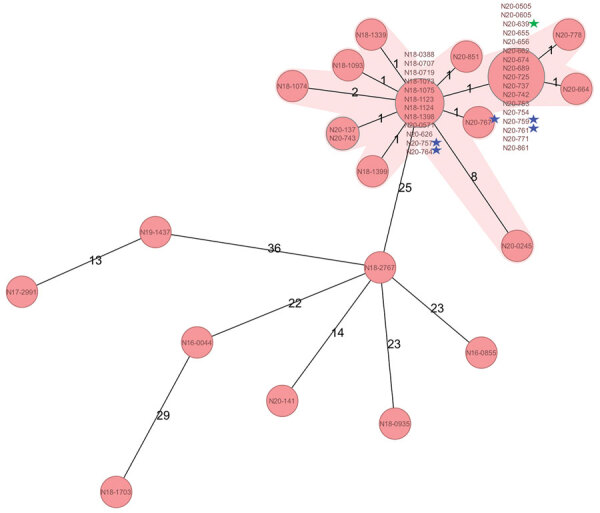
Minimum-spanning tree based on cgMLST allelic profiles of 34 human *Listeria monocytogenes* isolates, 1 food isolate, and 5 environmental isolates, Switzerland. Each circle represents an allelic profile based on sequence analysis of 1,701 cgMLST target genes. Values on connecting lines indicate number of allelic differences between 2 strains. Each circle contains the strain identification(s). The food isolate is indicated by a green star, and environmental strains are indicated by blue stars. Outbreak strains are shaded in pink and are shown in comparison with other *L. monocytogenes* sequence type 6 isolates from Switzerland collected during 2016–2020. cgMLST, core genome multilocus sequence typing.

A total of 22 strains grouped on the basis of WGS in a tight cluster, with the exception of N20–2045, which differed by >8 alleles (Figure 2). This strain was within the cluster definition. However, in absence of supportive epidemiologic data, we were not able to verify whether N20–0245 was truly involved in the outbreak.

Median age of the patients was 81 years (range <1–99 years). More than half of the patients were female (18/34, 53%). Of the 34 human isolates, 30 were from blood samples and 1 each from an abscess, ascites, maternal placenta tissue, or stool sample (Table). One case of perinatal transmission and 10 deaths (29%) were reported.

**Table:** *Listeria monocytogenes* 4b sequence type 6 cluster type 7448 isolates associated with listeriosis outbreak, Switzerland, 2018–2020*

Isolate ID	Date of isolation	Origin	Source	Patient age, y/sex	BioSample accession no.
N18–0388	2018 Mar 6	Human	Blood	82/F	SAMN15325567
N18–0707	2018 Apr 30	Human	Ascitic fluid	79/F	SAMN15325568
N18–0719	2018 May 2	Human	Blood	59/F	SAMN15325569
N18–1073	2018 Jun 26	Human	Blood	<1/F	SAMN15325570
N18–1074	2018 Jun 26	Human	Blood	88/F	SAMN15325571
N18–1075	2018 Jun 26	Human	Maternal placenta tissue	38/F	SAMN15325572
N18–1093	2018 Jun 27	Human	Blood	82/M	SAMN15325573
N18–1123	2018 Jul 3	Human	Blood	81/M	SAMN15325574
N18–1124	2018 Jul 3	Human	Blood	99/M	SAMN15325575
N18–1339	2018 Jul 24	Human	Blood	82/F	SAMN15325576
N18–1398	2018 Jul 31	Human	Blood	48/M	SAMN15325577
N18–1399	2018 Jul 31	Human	Blood	14/M	SAMN15325578
N20–0137	2020 Jan 22	Human	Blood	77/M	SAMN15325579
N20–0245	2020 Feb 7	Human	Blood	73/M	SAMN15325580
N20–0505	2020 Mar 17	Human	Blood	73/M	SAMN15325581
N20–0571	2020 Mar 30	Human	Blood	85/M	SAMN15325582
N20–0605	2020 Apr 6	Human	Blood	73/M	SAMN15325583
N20–0626	2020 Apr 15	Human	Blood	85/M	SAMN15325584
N20–0655	2020 Apr 20	Human	Blood	66/F	SAMN15325585
N20–0656	2020 Apr 20	Human	Blood	81/F	SAMN15325586
N20–0662	2020 Apr 22	Human	Blood	86/F	SAMN15325587
N20–0664	2020 Apr 22	Human	Blood	69/F	SAMN15325588
N20–0674	2020 Apr 23	Human	Blood	84/F	SAMN15325589
N20–689	2020 Apr 29	Human	Blood	63/F	SAMN15325590
N20–725	2020 May 4	Human	Blood	81/M	SAMN15325592
N20–737	2020 May 5	Human	Blood	86/M	SAMN15325593
N20–742	2020 May 6	Human	Blood	78/F	SAMN15325594
N20–743	2020 May 6	Human	Blood	37/M	SAMN15325595
N20–753	2020 May 8	Human	Blood	75/M	SAMN15325596
N20–754	2020 May 8	Human	Blood	85/F	SAMN15325597
N20–771	2020 May 11	Human	Blood	95/F	SAMN15325598
N20–778	2020 May 12	Human	Blood	95/F	SAMN15325599
N20–851	2020 May 22	Human	Perianal abscess	85/M	SAMN15325600
N20–861	2020 May 26	Human	Blood	83/F	SAMN15325601
N20–639	2020 Apr 30	Food	Cheese sample	NA/NA	SAMN15325591
N20–757	2020 May 3	Environment	Scrub sponge	NA/NA	SAMN15375881
N20–759	2020 May 3	Environment	Drainage channel	NA/NA	SAMN15375882
N20–761	2020 May 3	Environment	Door handle	NA/NA	SAMN15375884
N20–764	2020 May 3	Environment	Cellar floor	NA/NA	SAMN15375885
N20–767	2020 May 3	Environment	Ripening cellar floor	NA/NA	SAMN15375883

*ID, identification; NA, not applicable.

On April 30, 2020, a cheese manufacturer reported to the cantonal laboratory detection of *L. monocytogenes* from a sample of soft (brie) cheese made from pasteurized milk. Analysis had been conducted as part of the manufacturer’s routine quality control practices, which are mandatory in Switzerland (Swiss Foodstuffs Act, Article 23). The cheese isolate N20–639 matched the outbreak strain CT by WGS (Table; Figure 2). The cantonal authorities started the tracing of the distribution chain of the dairy. The cheese producer supplied several buyers who provide cheese to retailers throughout Switzerland. The buyers were requested to immediately stop the delivery of the products of this specific producer.

These findings prompted extensive environmental sampling on the production site of the manufacturer. A total of 50 swab specimens from locations, such as vats, cheese harps, skimming devices, sink drains, brushes, scrub sponges, trays, door handles, ripening cellar floors, and walls were obtained. Swabs were incubated in Half Frazer Broth (Bio-Rad, https://www.bio-rad.com) at 30°C for 48 h. *L. monocytogenes* was detected by real-time PCR with the Assurance Genetic Detection System (Endotell, https://www.endotell.ch) according to the manufacturer’s instructions. To obtain strains for WGS, 5 enriched Half Frazer Broth cultures were streaked on chromogenic *Listeria* agar plates (Oxoid, Pratteln, Switzerland) and incubated at 37°C for 24 h.

*L. monocytogenes* was identified in 11 (22%) of 50 environmental samples, and all 5 sequenced isolates matched the outbreak strain CT (Table; Figure 2). These results lead to a recall on May 5, 2020, of 26 items, including brie, sheep and goat cheese, and organic cheeses; production was stopped immediately. The findings were reported to the Epidemic Intelligence Information System for Food and Waterborne Diseases and Zoonoses. After the recall of the implicated products and a public warning issued by the Federal Food Safety and Veterinary Office, 7 cases of listeriosis caused by the outbreak strain were recorded (Figure 1). The last known case caused by this outbreak strain was sampled on May 20, 2020, and reported to SFOPH on May 25, 2020. Sequence data have been deposited in the National Center for Biotechnology Information (Bethesda, MD, USA) BioSample database under project no. PRJNA640586. We provide accession numbers (Table).

## Conclusions

This prolonged outbreak of *L. monocytogenes* 4b ST6 CT7448 caused 34 laboratory-confirmed listeriosis cases and 10 deaths. The outbreak investigation is an example of successful collaboration between laboratories and food safety and public health authorities to determine sources of contamination and reconstruct outbreak development. The results of the investigation implicated a cheese dairy with sanitation shortcomings and persisting environmental contamination throughout the production site. Isolation and WGS typing of *L. monocytogene*s from a quality-control cheese sample provided crucial information that enabled identification of the origin of contamination. WGS played a key role in showing close relatedness between the isolates from the cheese item and from the environment, and in linking the listeriosis cases from 2018 to the 2020 outbreak.

This outbreak highlights the risk for recontamination of pasteurized cheese products during manufacturing and emphasizes the need for routine sampling of products, manufacturing equipment, and the production environment. Routine quality controls should include WGS typing of environmental *L. monocytogenes* isolates to enable early recognition of potential food contamination and to ultimately mitigate the risk for listeriosis.
